# Transperitoneal vs retroperitoneal laparoscopic radical nephrectomy: a double-arm, parallel-group randomized clinical trial

**DOI:** 10.1186/s12894-023-01364-w

**Published:** 2024-02-03

**Authors:** Junyao Liu, Bin Zhang, Peng Qi, Xiaowei Ren, Duo Zheng, Yang He, Xu Zheng, Zhongjin Yue, Ye Li, Ningqiang Yang, Zhiping Wang, Junsheng Bao, Junqiang Tian, Li Yang, Zhenxing Zhai, Lingjun Zuo, Zizhen Hou, Jiaji Wang, Wei Wang, Hong Chang, Junhai Ma, Yunxin Zhang, Zhichun Dong, Zhilong Dong, Ganping Zhong, Hui Cheng, Pengyuan Lei, Zhongming Li, GongJin Wu, Panfeng Shang

**Affiliations:** 1 Department of Urology, Lanzhou University Second Hospital, No.82 Cui Ying Gate, Cheng Guan District, Lanzhou, 730030 Gansu China; 2 School of Public Health, Lanzhou University, Lanzhou, Gansu China; 3 Department of Urology, Xigu Branch of Lanzhou University Second Hospital, Lanzhou, Gansu China

**Keywords:** Renal cell carcinoma, Retroperitoneal approach, Transperitoneal approach, Laparoscopic radical nephrectomy

## Abstract

**Objective:**

To compare the outcomes of patients undergoing Retroperitoneal laparoscopic Radical nephrectomy (RLRN) and Transperitoneal laparoscopic Radical nephrectomy (TLRN).

**Methods:**

A total of 120 patients with localized renal cell carcinoma were randomized into either RLRN or TLRN group. Mainly by comparing the patient perioperative related data, surgical specimen integrity, pathological results and tumor results.

**Results:**

Each group comprised 60 patients. The two group were equivalent in terms of perioperative and pathological outcomes. The mean integrity score was significantly lower in the RLRN group than TLRN group. With a median follow-up of 36.4 months after the operation, Kaplan–Meier survival analysis showed no significant difference between RLRN and TLRN in overall survival (89.8% vs. 88.5%; *P* = 0.898), recurrence-free survival (77.9% vs. 87.7%; *P* = 0.180), and cancer-specific survival (91.4% vs. 98.3%; *P* = 0.153). In clinical T2 subgroup, the recurrence rate and recurrence-free survival in the RLRN group was significantly worse than that in the TLRN group (43.2% vs. 76.7%, *P* = 0.046). Univariate and multivariate COX regression analysis showed that RLRN (HR: 3.35; 95%CI: 1.12–10.03; *P* = 0.030), male (HR: 4.01; 95%CI: 1.07–14.99; *P* = 0.039) and tumor size (HR: 1.23; 95%CI: 1.01–1.51; *P* = 0.042) were independent risk factor for recurrence-free survival.

**Conclusions:**

Our study showed that although RLRN versus TLRN had roughly similar efficacy, TLRN outperformed RLRN in terms of surgical specimen integrity. TLRN was also significantly better than RLRN in controlling tumor recurrence for clinical T2 and above cases.

**Trial registration:**

Chinese Clinical Trial Registry (https://www.chictr.org.cn/showproj.html?proj=24400), identifier: ChiCTR1800014431, date: 13/01/2018.

**Supplementary Information:**

The online version contains supplementary material available at 10.1186/s12894-023-01364-w.

## Introduction

Renal cell carcinoma (RCC) constitutes approximately 3% of all cancers, with the highest incidence rates observed in western countries [1]. In most nations, the incidence rate of RCC continues to steadily increase [2]. This trend may be attributed to the amplified utilization of tomographic imaging techniques and longer life expectancies [2, 3].

For localized RCC, both radical nephrectomy (RN) and partial nephrectomy (PN) are curative treatment options recommended by clinical guidelines. Results from many studies [4–6] on RN and PN indicate that the surgical indications for PN appear to be expanding, which may somewhat overshadow the role of RN. Unfortunately, the only randomized controlled trial (RCT) comparing PN and RN was underpowered and limited to tumors smaller than 5 cm [4]. Consequently, EAU and AUA guidelines have been cautious about expanding the use of PN in recent years [7]. Currently, for T1b tumors, the EAU guidelines strongly recommend PN [8], while the AUA guidelines are relatively conservative and emphasize the need to consider contralateral renal function [9]. For clinical T2 tumors, RN remains the gold standard treatment and cannot be replaced.

Transperitoneal laparoscopic radical nephrectomy (TLRN) and retroperitoneal laparoscopic radical nephrectomy (RLRN) are the most commonly used surgical techniques for RCC treatment, each with its own advantages and limitations. However, current guidelines do not provide specific criteria for selecting between these approaches, and suggest that there is little technical difference between them.

Retrospective studies with large sample sizes have conducted detailed comparisons between TLRN and RLRN, and confirmed that perioperative and oncological outcomes are similar for both approaches [10, 11]. However, potential bias may exist due to substantial heterogeneity in stage distribution (T1 and T2) among these studies, and an increased tumor volume may account for the attenuation in technical benefits of RLRN. Considering that the study reports of two RCTs [12, 13] and one quasi-RCT [14] did not adhere to the CONSORT guidelines for reporting RCTs, the overall reporting quality is relatively low. Thus, we aimed to improve upon the limitations of previous research by designing a more robust study. The primary objective of our randomized controlled trial is to evaluate the effectiveness of RLRN compared to TLRN in patients with renal cancer today, taking into account both perioperative and oncological outcomes. Through this study, we hope to provide more definitive evidence on the relative merits of these two surgical approaches, which may help guide clinical decision-making in RCC treatment.

## Patients and methods

### Hypothesis, study design, and patients

This is a multicenter, double-arm, parallel-group randomized clinical study comparing TLRN and RLRN conducted in the Lanzhou University second hospital and Xigu branch of the Lanzhou University second hospital from January 2018 to May 2022.

According to the clinical study protocol, all patients older than 18 years and younger than 75 years, diagnosed as having localized RCC (stage T1-T3aN0M0) were screened for inclusion in the trial by the local clinical investigators. Exclusion criteria were patients with benign tumors or urothelial carcinoma of renal pelvic, renal vein and inferior vena cava invasion, patients with radiologically proven distant metastasis, history of other malignant tumors and/or chemotherapy, previous abdominal surgery, pregnancy and/or lactation, patients who were incapacitated, patients with disease classified as American Society of Anesthesiologists IV and V, T1 tumors on which PN could be performed, and refusal of the patient to sign the informed consent form.

The protocol followed the principles of the Helsinki declaration, was approved by the ethics committee of the second hospital of Lanzhou University (2017A-054) and was registered at Chinese Clinical Trial Registry (identifier: ChiCTR1800014431) on January 13, 2018.

### Randomization

Patients were randomly allocated to RLRN or TLRN in a ratio of 1:1. To avoid any unbalanced sample size between groups, we randomly allocated patients dynamically as per Pocock and Simon [15, 16], stratified by age (< 60, ≥ 60), clinical stage (T1, T2 + T3a) and typical symptoms (with, without). Minimization was accomplished with an algorithm based on stratification factors. After a patient was allocated, an imbalance index was calculated for both TLRN and RLRN approaches. The approach with the lower imbalance index was prioritized for future patient allocation. It should be mentioned that since the exclusion criteria included benign renal tumors and urothelial carcinoma of renal pelvic, we excluded these cases based on postoperative pathological reports. The imbalance index was then recalculated to determine the assignment for the next case.

### Interventions

Our LRN technique has been previously reported [17, 18]. Considering its well-established nature, this study will not elaborate on the details of LRN. All the surgeons had passed the LRN learning curve and completed 50 cases of each approach independently.

### Postoperative management and follow-up

All patients received standardized radical nephrectomy common clinical pathway [19]. Patients were re-examined at 3, 6, and 12 months after discharge, and then annually thereafter. According to the local protocol, abdominal ultrasound, blood biochemistry, and urine routine examination were performed at 3 months. Chest, abdominal, and pelvic CT scans were conducted at 6 and 12 months to monitor for signs of recurrence or other complications.

### Outcome measures

The data were collected from January 2018 to July 2023. The observations mainly included laparoscopic surgery time (defined as the time from placement of all trocars to resection of the entire kidney), total operative time, estimated blood loss (EBL), postoperative hospital stay (PLOS), incidence of adverse events (AEs), pathological outcomes, total hospital costs, and oncological outcomes. Of note, we established the specimen integrity [20] scoring tool, in which raters assessed the integrity of patient specimens according to the status of the Gerota fascia, perirenal fat envelope, and tumor capsule.

### Power calculations and statistical analysis

Sample size was estimated using data from published TLRN vs RLRN RCTs [12, 13], meta-analysis [21], and retrospective study based on our previous experience (unpublished). Using these data, we tested the difference in the laparoscopic operative time between the RLRN and TLRN groups. The true difference between the approaches was hypothesized to be 22.9 min with standard deviation of 34.2. To achieve 80% power, using an α error of 0.05(2-tailed) and an inflation rate of 10% to account for loss to follow-up, we required 40 participants per arm during the recruitment phase. Since there is no morally unacceptable risk in the main results of the analysis, there will be no planned interim analysis. All final analyses were based on intention-to-treat. Analysis of the primary endpoint was conducted according to per protocol.

Baseline characteristics were presented as frequencies (percentages) for categorical variables and mean (standard deviation, SD) or median (interquartile range, IQR) for continuous variables. Differences in distributions were compared between RLRN and TLRN groups using t-test or Wilcoxon rank-sum test for continuous variables and Fisher’s exact test or Pearson’s chi-square test for categorical variables as appropriate.

The Kaplan–Meier method was used to estimate overall survival (OS), recurrence-free survival (RFS), and cancer-specific survival (CSS). The Cox proportional hazards model was used to evaluate the prognostic risk factors in RFS and summarized as the hazard ratio (HR) and 95% confidence interval (CI). All results were considered statistically significant when two-sided *P*-values were < 0.05.

Statistical analysis was performed using SPSS 26.0 software (IBM Institute, Inc., Armonk, NY, USA) and R software, version 4.3.1 (R Project for Statistical Computing).

## Results

### Patient characteristics

**Fig. 1:**
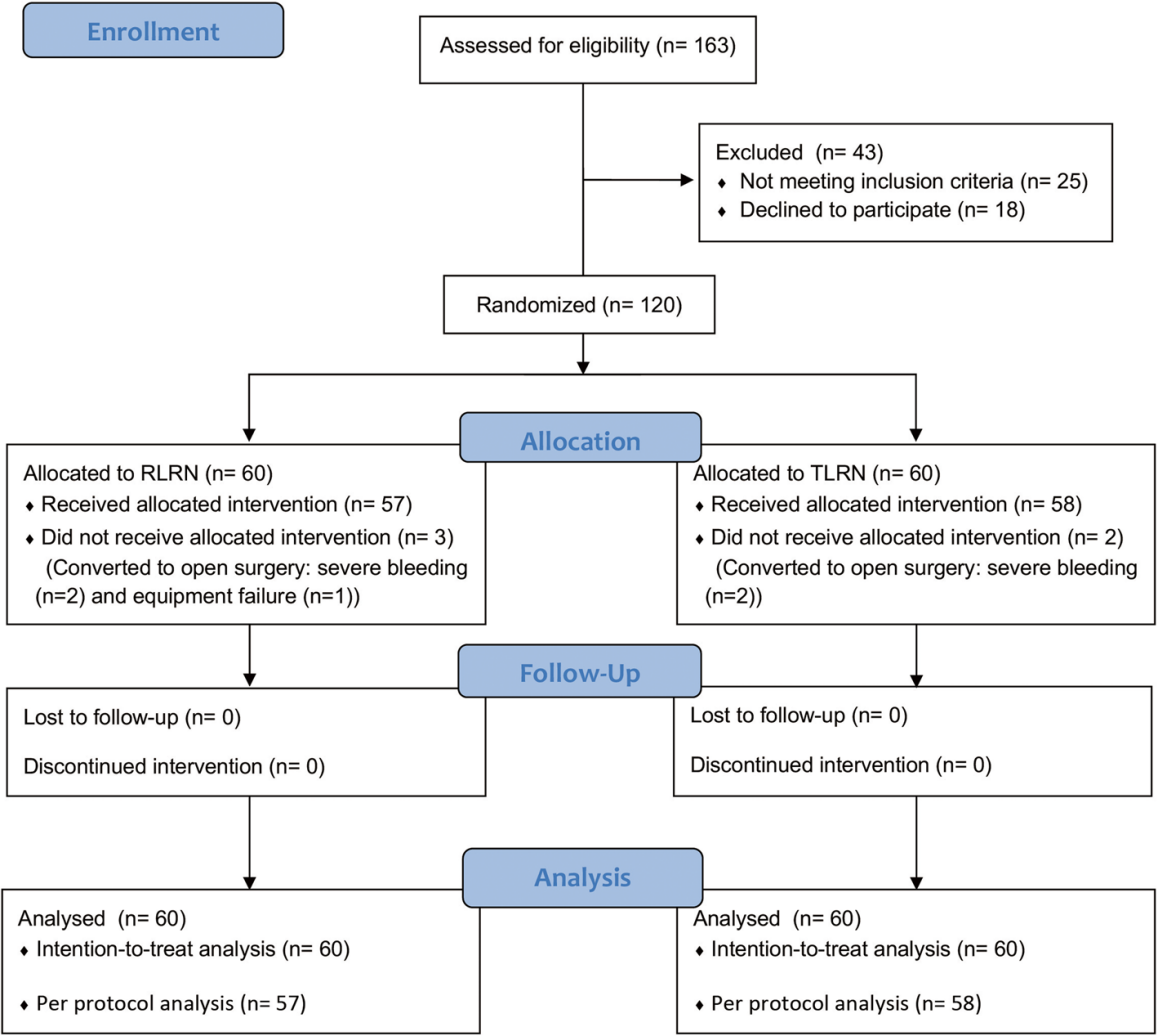
Trial flow chart. RLRN = retroperitoneal laparoscopic radical nephrectomy. TLRN = transperitoneal laparoscopic radical nephrectomy

Between 2018 and 2022, a total of 163 RCC patients were assessed for eligibility. Of 138 eligible patients (Fig. 1), 18 withdrew before randomization. Of 120 patients randomized, 60 were assigned to RLRN group (mean (SD) age, 57.4 (10.1) years and 36 (60%) male) and 60 were assigned to TLRN group (mean (SD) age, 57.0 (11.0) years and 39 (65%) male). Laparoscopic surgery was accomplished in 115 patients (95.8%); the remaining 5 (4.2%) were converted to open surgery. The main reason for conversion from laparoscopic to open procedure was intraoperative bleeding. Baseline characteristics, including clinical stage distribution (T1 or T2) and tumor size, were similar between treatment arms in the intent-to-treat population (Table 1).

**Table 1:** Demographic data and characteristics of patients who received RLRN or TLRN

*Characteristic*	*RLRN(n* = *60)*	*TLRN(n* = *60)*	*P* value
Age, yr, mean (SD)	57.4(10.1)	57.0(11.0)	0.822^b^
Male, n (%)	36(60)	39(65)	0.572^a^
BMI, kg/m^2^, median (IQR)	24.2(21.5 ~ 26.4)	23.7(21.3 ~ 26.5)	0.815^c^
ASA grade, n (%)			0.658^a^
ASA 1	2(3.3)	4(6.7)	
ASA 2	51(85)	48(80)	
ASA 3	7(11.7)	8(13.3)	
Any classic symptoms, n (%)	27(45)	28(46.7)	0.855^a^
Comorbidity, n (%)	27(45)	30(50)	0.583^a^
Posterior side, n (%)	23(38.3)	20(33.3)	0.568^a^
Left side, n (%)	30(50)	27(45)	0.583^a^
Location, n (%)			0.071^a^
Upper pole	8(13.3)	18(30)	
Upper-middle pole	12(20)	6(10)	
Mid pole	10(16.7)	14(23.3)	
Lower-middle pole	12(20)	6(10)	
Lower pole	18(30)	16(26.7)	
Clinical stage, n (%)			0.619^a^
cT1a	10(16.7)	6(10)	
cT1b	28(46.7)	31(51.7)	
cT2a	14(23.3)	12(20)	
cT2b	8(13.3)	11(18.3)	
Tumor size, cm, median (IQR)	6.1(5 ~ 7.6)	6.3(5 ~ 8.8)	0.472^c^

^a^Pearson’s χ^2^ test (or Fisher’s exact test)

^b^Independent sample t test

^c^Mann-Whitney U test

### Primary outcome

Overall, 115 patients underwent laparoscopic surgery. According to the per-protocol analysis, the median (IQR) laparoscopic operative time was similar in both groups (85 (61.5–116) min in RLRN group vs. 89 (69.5–120) min in TLRN group, *P* = 0.737) (Table 2).

**Table 2:** Perioperative and pathological data of patients who received RLRN or TLRN

*Characteristic*	*RLRN(n* = *60)*	*TLRN(n* = *60)*	*P* value
Laparoscopic operative time^d^, min, median (IQR)	85(61.5–116)	89(69.5–120)	0.737^c^
Total operative time, min, median (IQR)	150(117.5 ~ 180)	142.5(120 ~ 177.5)	0.945^c^
EBL, ml, median (IQR)	50(30 ~ 115)	100(32.5 ~ 200)	0.226^c^
Open conversion, n (%)	3(5)	2(3.3)	1.000^a^
Blood transfusion, n (%)	6(10)	4(6.7)	0.741^a^
Integrity score^e^, pts, mean (SD)	2.98(1.36)	4(1.34)	0.000^b^
Integrity score^e^, n(%)			0.009^a^
1	5(9.8)	1(1.9)	
2	18(35.3)	8(15.1)	
3	12(23.5)	8(15.1)	
4	8(15.7)	18(33.9)	
5	5(9.8)	9(17)	
6	3(5.9)	9(17)	
Pathology, n (%)			0.151^a^
ccRCC	56(93.3)	49(81.7)	
pRCC	1(1.7)	4(6.7)	
chRCC	1(1.7)	5(8.3)	
Other	2(3.3)	2(3.3)	
Pathological stage, n (%)			0.676^a^
pT1a	10(16.7)	6(10)	
pT1b	26(43.3)	29(48.3)	
pT2a	13(21.7)	10(16.7)	
pT2b	5(8.3)	8(13.3)	
pT3a	6(10)	7(11.7)	
Upstaging to pT3a, n (%)	6(10)	7(11.7)	0.769^a^
AEs, n (%)			0.746^a^
Vascular injury	5(8.3)	3(5)	
Liver injury	0(0)	1(1.7)	
Spleen injury	0(0)	1(1.7)	
Pleura injury	1(1.7)	0(0)	
Other	3(5)	2(3.3)	
Intake time, d, mean (SD)	2.18(0.70)	2.08(0.67)	0.426^b^
Time of drainage, d, mean (SD)	4.12(1.22)	4.13(1.47)	0.946^b^
PLOS, day, mean (SD)	6.78(2.09)	6.83(1.94)	0.892^b^
Total hospitalization charges, $, median (IQR)	3565.8 (3211.8 ~ 3927)	3536.5 (3229.8 ~ 4088.2)	0.704^c^

^a^Pearson’s χ^2^ test (or Fisher’s exact test)

^b^Independent sample t test

^c^Mann-Whitney U test

^d^Analysis in the per protocol population

^e^Analysis in 104 patients with integrity score

### Secondary outcomes

The median operative time was similar between the two groups (150 (117.5–180) vs. 142.5 (120–177.5) mins; *P* = 0.945). No significant differences were observed for EBL (50 (30–115) vs. 100 (32.5–200) ml; *P* = 0.226), conversion rate (5% vs. 3.3%; *P* = 1.000), PLOS (6.78 (2.09) vs. 6.83 (1.94) days; *P* = 0.892), or total hospitalization charges ($3565.8 ($3211.8-$3927) vs. $3536.5 ($3229.8-$4088.2); *P* = 0.704) between the two groups, as shown in Table 2.

Pathological outcomes, including case type distribution, pathological stage, and upstaging to pT3a, were similar between the two groups (Table 2). Final pathological review revealed upstaging to pT3a in 6 of 60 patients (10%) in the RLRN group and 7 of 60 patients (11.7%) in the TLRN group, with no significant difference observed between them.

Intra- and post-operative AEs were observed in 9 patients (15%) in the RLRN group and 7 patients (11.7%) in the TLRN group, with no significant difference between them (*P* = 0.746). The types of AEs observed were similar in both groups, with injury-related AEs being the most commonly identified (Table 2).

**Fig. 2:**
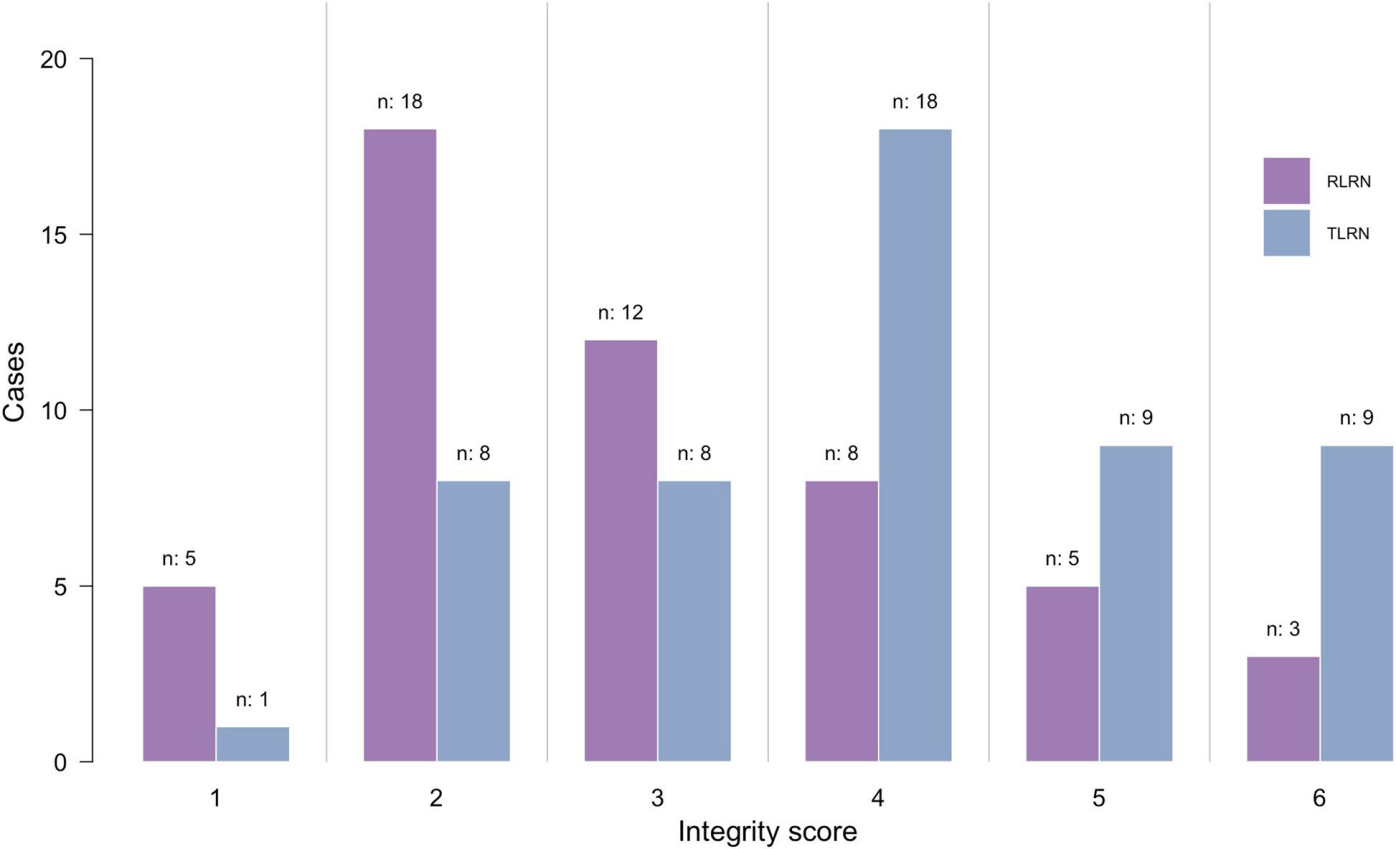
Distribution of the integrity score between RLRN group and TLRN group

In terms of integrity score, there was a significant difference in the distribution of integrity score between the two groups (Fig. 2). In RLRN group, most of the specimens were concentrated in 2-3points(2.98 ± 1.36), while in TLRN group, most of the specimens were concentrated in 4 points(4 ± 1.34). There was also a significant difference in mean point between the two groups (*p* < 0.001) (Table 2).

### Oncological outcomes

The RLRN group with a median follow-up of 34.6 (21.3 to 51) months, and the TLRN group with a median follow-up of 38.3 (24.6 to 47) months.During the follow-up period, recurrence was observed in a total of 19 (15.8%) patients, with 12( 20%) patients in the RLRN group and 7( 11.7%) patients in the TLRN group. Furthermore, a total of 10 patients (5 in each group) had died during the study period. Of these deaths, 5 patients (4 in the RLRN group and 1 in the TLRN group) were attributed to disease recurrence (Table 3).

**Table 3:** Oncological outcomes

**Total cohort**			
Variable	RLRN(*n* = 60)	TLRN(*n* = 60)	*P* value
Recurrence, n (%)	12(20)	7(11.7)	0.211^a^
Death, n (%)	5(8.3)	5(8.3)	1.000^a^
Cancer related death, n (%)	4(6.7)	1(1.7)	0.361^a^
Time of follow-up, mo, median (IQR)	34.6(21.3–51)	38.3(24.6–47)	0.503^b^
**T1 subgroup**			
Variable	RLRN(*n* = 38)	TLRN(*n* = 37)	*P* value
Recurrence, n (%)	1(2.6)	2(5.4)	0.981^a^
Death, n (%)	2(5.3)	2(5.4)	1.000^a^
Cancer related death, n (%)	1(2.6)	0(0)	1.000^a^
**T2 subgroup**			
Variable	RLRN(*n* = 22)	TLRN(*n* = 23)	*P* value
Recurrence, n (%)	11(50)	5(21.7)	**0.048**^**a**^
Death, n (%)	3(13.6)	3(13)	1.000^a^
Cancer related death, n (%)	3(13.6)	1(4.3)	0.568^a^

^a^Pearson’s χ^2^ test (or Fisher’s exact test)

^b^Mann-Whitney U test

**Fig. 3:**
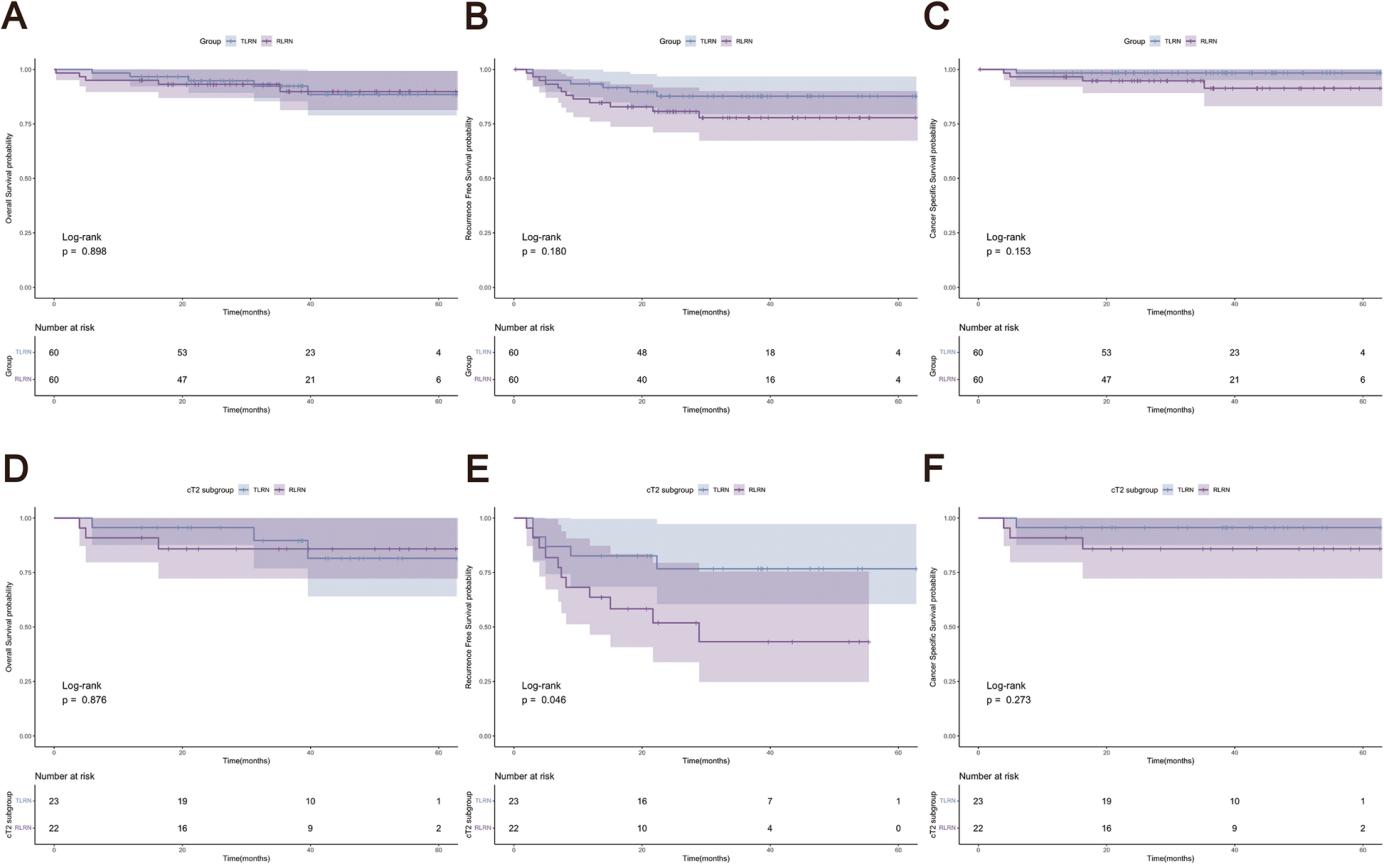
Kaplan–Meier estimates of (**A**) overall, (**B**) recurrence-free, and (**C**) cancer-specific survival according to surgical approach. Kaplan–Meier estimates of (**D**) overall, (**E**) recurrence-free, and (**F**) cancer-specific survival in cT2 subgroup according to surgical approach

Kaplan–Meier survival estimates were used to calculate the survival probabilities from surgery to death or last follow-up. No statistically significant differences were observed between the two groups in terms of OS, RFS, and CSS, as shown in Fig. 3A-C.

Stage-specific oncological outcomes were analyzed by surgical approach. Statistically significant differences existed in recurrence rate of patients with cT2 tumor (Table 3) and in RFS of patients with cT2 tumor (Fig. 3D-F) between the two groups.

The multivariate Cox regression analysis revealed that RLRN (HR: 3.35; 95%CI: 1.12–10.03; *P* = 0.030), male (HR: 4.01; 95%CI: 1.07–14.99; *P* = 0.039), and tumor size (HR: 1.23; 95%CI: 1.01–1.51; *P* = 0.042) were independent risk factors for tumor recurrence of patients with cT2 tumor (Supplementary Table 1).

## Discussion

To our knowledge, this trial is the first to prospectively and comprehensively compare outcomes of RLRN and TLRN while following CONSORT guidelines. The study was powered on laparoscopic operative time for three reasons. Firstly, operative time reflects the complexity of the surgery or technical difficulties encountered during the procedure. Secondly, unpredictable intraoperative AEs can result in delays in operative time. Thirdly, compared with total operative time, laparoscopic operative time is more objective and can help control bias caused by technical reasons during the procedure. It was expected that the perioperative performance of the two approaches may differ from other early studies. However, this study did not find any significant differences in common surgical evaluation indicators, including the primary outcome, between well-matched groups with similar RCC types. These findings are consistent with other series of studies [13, 14, 22–24]. An issue worth noting is that although this comparison has been done numerous times in the literature, in some studies where the proportion of low-stage tumors is relatively high, it has indeed been demonstrated that RLRN has a shorter operative time [12]. However, does faster operation necessarily mean better outcomes?

During clinical practice, we have observed that RLRN's limited working space becomes more pronounced when dealing with larger tumor volumes. Furthermore, tumors with higher clinical stages, particularly those with T3 upstaging, often display wider intertissue adhesion and increased vascular branching. As a result, achieving optimal resection can be a challenge in such cases. Surgeons may incise Gerota's fascia during RLRN to maintain peritoneal integrity and minimize bleeding, mobilizing the kidney in the avascular space between the perirenal fat and anterior renal fascia. This approach, however, can make it difficult to remove some parts of Gerota's fascia, particularly the anterior renal fascia. Deger et al. discovered that retroperitoneoscopic approach made it difficult to remove the entire tumor, including the perirenal tissue covered by Gerota's fascia, especially in T2 and T3a tumors [25]. In Taue et al.'s study, most patients underwent RLRN, and their kidney was dissected and removed within Gerota's fascia [24]. This does not adhere to the resection principles of radical nephrectomy and is likely to adversely impact tumor control, particularly with regards to the prognosis of stage T3a renal cancer with tumor invasion of Gerota's fascia. It has been suggested that performing a perifascial nephrectomy is crucial for preventing local recurrence after surgery since around 25% of clinical T1b/T2 RCCs manifest perirenal fat involvement [26, 27]. If intrafascial resection is prevalent in institutions performing RLRN, the risk of local residual may increase, which could result in some patients developing advanced renal cell carcinoma. In the current study, we found that specimen integrity in RLRN was significantly inferior to TLRN. We believe that in specific cohorts, this difference may be amplified.

Oncological outcomes are an essential consideration when choosing between TLRN or RLRN. It has been reported that almost 30% of patients experience relapse after treatment [28]. In our study, this proportion was 15.8%. Ha et al. found comparable recurrence rates between the two approaches (4.2% vs. 1.9%) after a follow-up period ranging from 2 to 93 months [10]. In the previous RCTs, one study [12] exclusively reported one case of bone metastasis in TLRN group, while another study [13] identified four cases of recurrence (3 in TLRN group and 1 in RLRN group) and six cases of mortality (2 in TLRN group and 4 in RLRN group). Our results were consistent with these studies, with no difference in OS, RFS, and CSS between the two approaches. However, when we stratified analyses according to clinical stage, the results changed. In the cT2 subgroup, disease recurrence was found in 11 of 22 (50%) patients managed with RLRN and in 5 of 23 (21.7%) patients treated with TLRN, at a mean follow up period of 34.6 and 38.3 months, respectively. Kaplan–Meier analyses showed a lower RFS in patients treated with TLRN than with RLRN. Additionally, the conclusion that the integrity score of RLRN group was lower than that of TLRN group still held in this subgroup, indicating a possible association between specimen integrity and disease recurrence. We further reviewed the literature to support our findings and found most studies did not report stage-specific oncological outcomes by surgical approach. Only one study reported comparable 5-year disease-free survival between TLRN and RLRN in T2 stage cases (78.4% vs. 90%). Furthermore, current comparisons for clinical T2 and higher stage RCC are primarily focused on PN versus RN and open versus laparoscopy. There is a scarcity of studies on TLRN versus RLRN, making it difficult to compare our findings. From the limited literature available, we found that the upper limit of long-term recurrence rates for RN/LRN treatment in T2 and higher stage RCC ranges from 23.5% to 58% [29–32]. Hence, despite the seemingly high recurrence rate when compared to reports from other LRN populations, it is still within the range documented in the literature.

This study has several limitations. Firstly, due to the fact that only two centers ultimately participated in the trial and there were not many surgeons proficient in TLRN, the recruitment period was long. Secondly, the study included a relatively small heterogeneous cohort, which raises concerns about statistical power. To address this issue, we increased the sample size beyond the calculated number. Finally, neither patients nor surgeons were blinded, which could introduce potential bias. Despite these limitations, this randomized controlled trial has the largest sample size and longest follow-up period to date in the comparison of RLRN and TLRN.

## Conclusions

Our study showed that although RLRN versus TLRN had roughly similar efficacy, TLRN outperformed RLRN in terms of surgical specimen integrity. However, in terms of tumor control within the cT2 subgroup, RLRN was significantly inferior to TLRN.

### Supplementary Information

**Additional file 1: Supplementary Table 1. **Univariate and multivariate analysis of recurrence-free survival in patients with clinical T2 stage RCC.

## Data Availability

J.Y.L and B.Z had full access to all the data in the study and take responsibility for the integrity of the data.

## Abbreviations

LRN: Laparoscopic Radical nephrectomy
RLRN: Retroperitoneal laparoscopic radical nephrectomy
TLRN: Transperitoneal laparoscopic radical nephrectomy
RCC: Renal cell carcinoma
RCT: Randomized controlled trial
EBL: Estimated blood loss
PLOS: Postoperative length of stay
AEs: Adverse events
OS: Overall survival
RFS: Recurrence-free survival
CFS: Cancer-specific survival
