# Supplementary material for: Transperitoneal vs retroperitoneal laparoscopic radical nephrectomy: a double-arm, parallel-group randomized clinical trial
Source: BMC Urol. 2024 Feb 3;24:29. doi: 10.1186/s12894-023-01364-w (PMC10838419; doi:10.1186/s12894-023-01364-w)
Supplement: Supplementary file 1 — Additional file 1: Supplementary Table 1. Univariate and multivariate analysis of recurrence-free survival in patients with clinical T2 stage RCC. [file 12894_2023_1364_MOESM1_ESM.docx]

Supplementary Table 1. Univariate and multivariate analysis of recurrence-free survival in patients with clinical T2 stage RCC

|  | *Univariate regression analysis* | | |  |  | *Multivariate regression analysis* | | | |
| --- | --- | --- | --- | --- | --- | --- | --- | --- | --- |
| *Variables* | *Hazard ratio* | *95%CI* | P |  | B | | *Hazard ratio* | *95%CI* | P |
| Operative approach |  |  |  |  |  | |  |  |  |
| RLRN | 2.80 | 0.97-8.11 | 0.057 |  | 1.21 | | 3.35 | 1.12-10.03 | **0.030** |
| TLRN | 1 (ref) |  |  |  |  | | 1 (ref) |  |  |
| Age | 1.02 | 0.98-1.07 | 0.352 |  | 0.04 | | 1.04 | 0.98-1.10 | 0.233 |
| Male | 3.18 | 0.90-11.27 | 0.072 |  | 1.39 | | 4.01 | 1.07-14.99 | **0.039** |
| Body mass index | 0.95 | 0.83-1.08 | 0.389 |  |  | |  |  |  |
| ASA | 1.37 | 0.56-3.36 | 0.488 |  |  | |  |  |  |
| Tumor size | 1.10 | 0.92-1.30 | 0.295 |  | 0.21 | | 1.23 | 1.01-1.51 | **0.042** |
| Posterior side | 1.56 | 0.57-4.31 | 0.388 |  |  | |  |  |  |
| Left side | 0.59 | 0.22-1.57 | 0.288 |  |  | |  |  |  |
| Tumor location |  |  | 0.663 |  |  | |  |  |  |
| Upper pole | 1 (ref) |  |  |  |  | |  |  |  |
| Upper-middle pole | 3.07 | 0.62-15.23 | 0.171 |  |  | |  |  |  |
| Mid pole | 2.27 | 0.38-13.62 | 0.370 |  |  | |  |  |  |
| Lower-middle pole | 1.50 | 0.21-10.68 | 0.687 |  |  | |  |  |  |
| Lower pole | 2.88 | 0.48-17.26 | 0.247 |  |  | |  |  |  |
| Histological type |  |  | 0.121 |  |  | |  |  |  |
| Clear cell | 1 (ref) |  |  |  |  | |  |  |  |
| Papillary | 1.03 | 0.13-7.93 | 0.981 |  |  | |  |  |  |
| Chromophobe | 0.70 | 0.91-5.43 | 0.734 |  |  | |  |  |  |
| Other | 6.40 | 1.35-30.23 | **0.019** |  |  | |  |  |  |
| Fuhrman grade | 1.50 | 0.71-3.16 | 0.286 |  |  | |  |  |  |

The significance of bold values represent P < 0.05
